# Synergetic Effects of K, Ca, Cu and Zn in Human Semen in Relation to Parameters Indicative of Spontaneous Hyperactivation of Spermatozoa

**DOI:** 10.1371/journal.pone.0152445

**Published:** 2016-03-31

**Authors:** Ivan Bolanca, Jasmina Obhodas, Dejan Ljiljak, Lidija Matjacic, Krunoslav Kuna

**Affiliations:** 1 University Hospital Centre “Sestre milosrdnice”, Department of Obstetrics & Gynecology, Vinogradska c. 29, 10000 Zagreb, Croatia; 2 Ruder Boskovic Institute, Bijenicka c. 54, 10000 Zagreb, Croatia; Cinvestav-IPN, MEXICO

## Abstract

We have observed that sperm quality parameters indicative of spermatozoa hyperactivation such are lower “linearity” and “straightness”, and as showed by this research “elongation”, were more pronounced in patients with normal spermiogram compared to the group of men with reduced sperm motility who were undergoing routine *in vitro* fertilisation. The research encompassed 97 men diagnosed with normozoospermia (n = 20), asthenozoospermia (n = 54) and oligoasthenozoospermia (n = 23). The findings indicate that sperm quality of patients with normal spermiogram diagnosed according to WHO criteria, may be compromised by showing premature spontaneous hyperactivation which can decrease the chances of natural conception. We assessed synergistic effects of multiple chemical elements in ejaculated semen to find if premature spontaneous hyperactivation of spermatozoa can be a sign of imbalanced semen composition especially of elements K, Ca, Cu and Zn. Human semen samples showing low or high baseline status of chemical elements concentrations were found in samples from all three diagnostic groups. However, correlation of K/Ca and Cu/Zn ratios, taking into account samples from all three groups of men, were negative at statistical significance level p = 0.01. We tested if the negative correlation between K/Ca and Cu/Zn ratio works for greater number of semen samples. We found the negative correlation to be valid for 175 semen samples at statistical significance of p = 0.00002. The ratio of K/Ca and Cu/Zn, i.e. increased concentrations of K and Zn in comparison to concentrations of Ca and Cu, were associated with a decrease of “straightness” in the group of men with normal spermiogram and pronounced spontaneous hyperactivation of spermatozoa, implying that these elements act in synergy and that the balance of elements and not their absolute concentrations plays the major role in premature spermatozoa hyperactivation in ejaculated semen.

## Introduction

Motility is the most essential sperm function which in large extent reflects the reproductive health of man. Sperm matures and obtains motility after major physiological modifications as a result of sequential interactions between transiting male gamete and secretions of the male reproductive system. The precise nature of these interactions is a subject of a longstanding interest in an effort to fully understand the elaborate process of fertilization. Hyperactivated motility is a deviation from the relatively linear and progressive spermatozoa swimming pattern. As a part of sperm capacitation, it is characterised by asymmetrical flagellar beating caused by an increase in the amplitude of the principal flagellar bend [1]. It is generally believed that random changes of swimming directions as well as high velocity, but low progression characteristic of hyperactivation most likely help sperm to find direction through the female reproductive tract toward the egg guided by thermotaxis, rheotaxis and chemotaxis [2], to increase chances to get detached if stuck to the oviductal epithelium [2] and to penetrate the highly viscous extracellular matrix of the egg [3]. According to Mortimer *et al*. [4] spermatozoa are classified as hyperactivated if demonstrating high “curvilinear velocity” (VCL), high “amplitude of lateral head displacement” (ALH) and low “linearity” (LIN). Mortimer *et al*. [4] defined criteria of hyperactivated spermatozoa as VCL ≥150 μm/s, ALH≥7 μm and LIN≤50%, whereas De Lamirande and Gagnon [5] criteria for hyperactivated spermatozoa were VCL≥80 μm/s, ALH≥6.5 μm and LIN≤65%. Low “straightness” (STR) is another motility parameter indicative of sperm hyperactivation. This research showed that low “elongation”, a morphological sperm quality parameter calculated as sperm head width over sperm head length, is associated to sperm hyperactivation. Sperm from semen ejaculates under normal circumstances should not exhibit spontaneous hyperactivation [6]. Hyperactivated sperm is normally observed in female tract i.e. incubated under capacitating conditions. The presence of spontaneous hyperactivation in ejaculate is considered premature [5].

Ejaculated sperm is suspended in seminal plasma, a complex fluid made of secretions from the rete testis, epididymis, ampulla of ductus deferens, seminal vesicles, prostate, urethral and bulbo-urethral gland [7]. The constituents from these secretions sequentially interact with sperm during its transit before and at the ejaculation and are responsible for obtaining full sperm functioning. They also provide a nutritive-protective medium for spermatozoa and are important in stimulation and preparation of the female reproductive tract for successful sperm transport and eventual implantation [8].

Some of the most essential elements for sperm maturation and capacitation are K, Ca, Cu and Zn. The major source of K, Ca and Zn in semen is prostate [9, 10], although these elements as well as Cu are found in all secretions from the male reproductive tract. Semen contains higher concentrations of antioxidants and Zn than any other biological fluid [11, 8]. One of the most active antioxidant enzyme in semen is copper-zinc superoxide dismutase (CuZnSOD) [8], a major source of Cu concentration in seminal plasma. The CuZnSOD source is mostly related to seminal vesicle [8]. In the normal course of events spermatozoa are expelled in the first third of ejaculate together with the prostatic fluid [12]. Prostatic fluid is rich in citrate, thus providing a slightly acidic environment and sustained quantity of bio-available K, Ca and Zn in form of cations [9]. The last two thirds of ejaculate contain mainly seminal vesicular fluid which increases pH of semen causing increased citrate chelation of zinc [12]. Seminal vesicular fluid is also rich in high molecular weight Zn ligands [12]. It is possible, however, to conclude that in such environment K and Ca ions would be also chelated into complex molecules [9]. Adding high quantities of CuZnSOD through seminal vesicular fluid also plays an important role in preventing hyperactivation. De Lamirande & Gagnon [13] showed that SOD hindered premature spermatozoa hyperactivation after ejaculation by preventing overproduction of reactive oxygen species (ROS). Sustained levels of ROS, as well as bio-available K, Ca and Zn will still be required for forward progression induction, whereas activation of sperm forward progression is prerequisite for sperm hyperactivation.

Zn is reversible removed from the spermatozoa thiol groups of cysteine, the amino acid found in high amounts in spermatozoa chromatin [12] and outer dense fibers (ODF) [14]. Zn in chromatin and ODF forms salt bridges with thiols [12, 14], thus preventing oxidation of thiols into disulfide bridges. Formation of disulfide bridges by Zn removal stabilizes and stiffens the chromatin and ODF structures [12, 14]. Both, chromatin and ODF, are designed to easily incorporate and release Zn ion depending on the external environment [12, 14]. ODF stiffening allows small flagellar wave amplitudes characteristic for progressive motility which is crucial for transferring spermatozoa through the female reproductive tract. Contrary, the high amount of Zn incorporated in spermatozoa ODF causes softening of its consistency leading to nonlinear (hyperactivated) motility. Incorporation of Zn in chromatin results in elongated, thinner sperm head. The absence of the transformation of round spermatozoa into elongated ones is the earliest sign of Zn deficiency in rats’ testis [15]. Another role of Zn in semen may be to prevent destruction of DNA in spermatozoa by inhibition of the DNase as suggested by Quinn [16], who showed that addition of Zn and citrate to ram seminal plasma reduced the activity of DNase. The Zn from prostatic fluid may also exhibit inhibitory effect upon activity of CuZnSOD enzymes. Results presented by Gavella *et al*. [17] showed that increase in Zn resulted in concentration-dependent decrease of SOD-like activity in human spermatozoa. This mechanism enables a greater ROS activity necessary for motility induction, both progressive and hyperactivated. Considerably recent evidence suggest that submicromolar concentrations of free Zn^2+^ hyperpolarizes sea urchin sperm membrane potential by activation of a K+ channel (Slo3) and increase the concentration of intracellular Ca^2+^ [18]. It is possible that mammalian sperm also possess the similar mechanism of Ca^2+^ entry. Lishko *et al*. [19] showed that removal of external Zn^2+^ activates voltage-gated proton channel Hv1 characteristic for human spermatozoa.

Ca ions in interplay with K ions are essential in signal transduction mechanisms and in mammalian sperm flagellar movement regulation. CatSper channels are responsible for Ca ion influx whereas the rise in intracellular pH precedes an influx of Ca ions [3, 20]. Extracellular K ion in alkaline media is responsible for intracellular sperm alkalization (pHi) presumably as a consequence of membrane depolarization [21]. Membrane depolarization in alkaline media enables activation of Hv1 channels [22]. It was observed that efflux of K ions through sperm-specific K+ channel (Slo3) by evoking membrane hyperpolarization enables Ca ions entry through a specific Ca ion channel CatSper [3, 23]. Activation of Slo3 channels could be a driving force for Na^+^/H^+^ exchange (NHE) or other Na^+^-dependent mechanisms controlling pHi that precedes the activation of Ca influx [23]. Thus, it is possible that human sperm may utilize two mechanisms for proton efflux (intracellular sperm alkalization). One involves activation of Hv1 through membrane depolarization and another activation of NHE mechanism through membrane hyperpolarization [23].

Both ROS and Ca influx are required to activate sperm motility. However, too high ROS concentration and higher Ca influx will lead to premature sperm hyperactivation in ejaculate. Even higher levels of ROS may eventually induce sperm cell death [24]. In addition, the spermatozoa will arrest if they are exposed to too high K concentrations [1]. One of the possible causes may be the Ca overload [3]. Another explanation is that too high concentrations of K ions inhibit enzymes responsible for fructolysis [25]. It is known that K is a natural metabolic inhibitor [8]. Higher concentration of K in seminal plasma decreases sperm metabolism, thereby decreasing sperm motility [8]. It was shown by Singh *et al*. [25] that K, together with Al and sulphate ion from aluminium potassium sulphate spermicide (potash alum), arrests sperm in time depending on spermicide concentration.

The above described mechanisms show the importance of K, Ca, Cu and Zn in obtaining and maintaining sperm motility. These elements create a homeostatically regulated environment in semen through their synergistic effects. Present paper shows that imbalance in their concentration can be associated to occurrence of spontaneous premature hyperactivation in ejaculates. Additional elements that have been analyzed in ejaculates by using Energy-dispersive x-ray fluorescence (EDXRF) were Fe, Ni, As, Br, Sr, Y and Pb, whereas concentrations of As and Pb were below the Minimum Detection Limit (MDL) of 0.10 μg/ml in all samples.

## Materials and Methods

### Study population

Ninety seven male partners in infertile couples attending the reproduction unit of “Sestre milosrdnice” University Hospital, Zagreb, Croatia, diagnosed with normozoospermia (n = 20), asthenozoospermia (n = 54) and oligoasthenozoospermia (n = 23) according to WHO criteria were recruited for this study. Participants were asked to provide their written informed consent to participate in the study. The procedure and all data collection were approved by the Ethical committee of the University Clinical Hospital Center “Sestre milosrdnice”, Zagreb (Croatia), the Ethical committee of the University Clinical Hospital Center Rijeka, Rijeka (Croatia) and the Medical faculty of the University of Rijeka (Croatia). A survey questionnaire included questions on age, marital status, occupation, alcohol consumption, body mass index, smoking status and other patient data.

### Semen analysis

Semen samples were collected by masturbation after 3–5 days of recommended abstinence from sexual intercourse or masturbation. Samples kept at room temperatures were brought to the laboratory within 1 hour of collection. Aliquots were taken after liquefaction at 37°C. Sperm motility of 97 patients who were encompassed by this study was analysed by using CASA (Hamilton Thorne, IVOS II, Beverly, USA) in accordance to the World Health Organization guidelines [26]. The acquisition frequency was 60 Hz, tracking time 2 s and field of view 300 x 300 μm. 200 spermatozoa were analysed per sample in dual 10 μm deep counting chambers. Minimum thresholds were set for curvilinear velocity and lateral head movement and a maximum threshold for path linearity. Samples with sperm concentration greater than 50 million/mL were diluted before CASA analysis with non-capacitating sperm wash medium produced by ORIGIO Ltd. The control sample was analysed by CASA and by manual evaluation as a part of the QC procedure.

Following semen-quality parameters were analysed by using CASA: volume (ml), sperm concentration (M/ml), “motility” (%), “progressivity” (%), “rapidity” (%), average path velocity (VAP; μm/s), straight-line velocity (VSL; μm/s), curvilinear velocity (VCL; μm/s), amplitude of lateral head displacement (ALH; μm), Beat Cross Frequency (BCF; Hz), “straightness” (STR; % = (VSL/VAPx100)), “linearity” (LIN; % = VSL/VCLx100), “elongation” (ratio of sperm head width to sperm head length, %) and “head surface area” (sperm head width x sperm head length, μm*2). The remaining samples were stored at -80°C for further analysis. In the absence of men proven recent fertility, normal semen (normozoospermia) in this study was defined by the WHO criteria. The WHO 2010 criteria for normozoospermia (N) were as follows: volume ≥ 1.5 ml, concentration ≥ 15 million/ml, total number of spermatozoa ≥ 39 million, total motility ≥ 40%, progressive motility ≥ 32%, vitality ≥ 58% and normal morphology ≥ 4%. Asthenozoospermia (A) was defined as having percentages of progressively motile spermatozoa below the lower reference limits. Oligoasthenozoospermia (OA) was defined as having a concentration of spermatozoa and percentages of progressively motile spermatozoa below the lower reference limits.

After liquefaction, a 250-μl aliquot was transferred into 2-ml Eppendorf snap-cap tubes and stored at -80°C for chemical elements analysis. Samples were defrosted to add 15 μg of gallium as an internal standard, then frozen again in liquid nitrogen and lyophilised overnight by Labconco FreeZone Pluse 2.5L Cascade Lyophilizer. Lyophilised samples were patched on 2.5-cm Millipore filters and analysed by using EDXRF. The EDXRF technique has several advantages in sperm analysis over other analytical methods; it requires minimum sample preparation which helps to prevent contamination, it is non-destructive and elements can be analysed simultaneously in a relatively large (representative) sample volume (250 μl). Measurements were carried out using X-ray tube with W anode, Mo secondary target and Canberra Si(Li) detector (FWHM = 175 eV) in orthogonal geometry with working parameters of 35 kV and 35 mA. Samples were irradiated for 1000 s. Collected x-ray spectra were fitted with the IAEA AXIL program, QXAS software package, using energies of the principal K_α_ emission lines. A standard addition method was applied in quantitative analysis of K, Ca, Fe, Ni, Zn, Cu, As, Br, Sr, Y and Pb concentrations. TraceCERT-certified solutions were used for calibration and for quality control/ quality assurance (QC/QA). Minimum detection limit (MDL) were 1.00 μg/ml for K and Ca; 0.70 μg/ml for Fe; 0.40 μg/ml for Ni and Cu and 0.10 μg/ml for Zn, As, Br, Sr, Y and Pb. The resulting concentrations had following errors: K: 2.29%, Ca—17.46%, Fe—1.60%, Ni—5.98%, Cu: 7.61%, Zn: 11.89%, Br: 3.51%, Sr: 8.85%, Y: 2.27%.

Additional 78 samples of semen collected from men attending the reproduction unit of “Sestre milosrdnice” University Hospital in Zagreb were analysed by EDXRF, however the sperm quality was analysed by manual assessment procedures with respect to sperm concentration and forward progression parameters.

### Statistical analysis

Descriptive statistics, Spearman correlation coefficients (rank-order non-parametric test) and Pearson correlation coefficients, Kruskal-Wallis test (KW) and Principal Component Analysis (PCA) were used to evaluate results with respect to concentrations of chemical elements, age, abstinence and semen-quality parameters. Spearman correlation coefficients were evaluated for p≤0.05 (*r* critical = ±0.20), p≤0.01 (*r* critical = ±0.26), p≤0.001 (*r* critical = ±0.33) and p≤0.0001 (*r* critical = ±0.41). All statistical analyses were performed by using Statistica 6.0 software. Post hoc analyses Mann-Whitney U test and Tukey HSD were used to determine which medians differed when KW indicated a significant difference in parameters STR, LIN, “elongation”, VCL and ALH among N, A and OA groups of patients.

## Results

### Descriptive statistics

The descriptive statistics are reported in Table 1. In all samples, the concentrations of As and Pb were below the MDL of 0.10 μg/ml, suggesting that research subjects were not exposed to significant levels of these toxic elements.

**Table 1:** Descriptive statistics for 97 semen samples.

	Mean	Med.	Min.	Max.	Lower Q.	Upper Q.	Std.Dev.	Skew.	Kurt.
**K μg/ml**	4210.8	3797.8	223.9	19688.9	2405.3	5327.0	2894.8	2.11	7.99
**Ca μg/ml**	877.9	788.5	104.7	3564.9	513.3	1092.3	553.7	1.65	5.00
**Fe μg/ml**	20.31	14.28	2.66	79.74	10.05	23.65	15.94	1.67	2.72
**Ni μg/ml**	2.68	2.34	0.71	7.67	1.61	3.35	1.46	1.25	1.58
**Cu μg/ml**	7.54	6.27	0.99	23.12	5.07	9.97	4.14	1.17	1.75
**Zn μg/ml**	96.80	77.98	20.95	423.89	50.02	123.74	66.93	1.94	5.45
**Br μg/ml**	0.58	0.30	0.10	4.22	0.10	0.63	0.84	2.86	8.42
**Sr μg/ml**	0.25	0.19	0.02	1.07	0.10	0.37	0.18	1.39	3.22
**Y μg/ml**	1.51	1.34	0.25	4.45	1.05	1.83	0.79	1.24	2.27
**Age**	35	34	22	54	32	38	5.8	0.62	1.34
**Abst. (days)**	4.2	4.0	2.0	7.0	3.0	5.0	1.2	0.72	-0.14
**Vol. (ml)**	3.3	3.3	0.8	7.8	2.2	4.0	1.5	0.67	0.40
**Conc.(M/ml)**	49.9	31.5	0.3	237.7	16.3	64.8	51.9	1.93	3.61
**Motility (%)**	25.9	17.0	0.0	90.0	3.0	43.0	26.1	0.87	-0.54
**Progressive (%)**	14.5	8.0	0.0	50.0	2.0	28.0	14.6	0.73	-0.86
**Rapid (%)**	21.9	12.0	0.0	85.0	2.0	39.0	23.5	0.92	-0.43
**VAP (mm/s)**	41.7	44.2	0.0	78.9	34.2	54.5	18.0	-0.87	0.63
**VSL (mm/s)**	35.2	39.3	0.0	62.6	29.8	44.5	14.8	-1.04	0.78
**VCL (mm/s)**	56.6	59.0	0.0	114.6	45.1	75.8	25.0	-0.69	0.53
**ALH (mm/s)**	2.21	2.20	0.00	8.50	1.40	3.00	1.32	0.82	4.33
**BCF (Hz)**	11.1	11.9	0.	36.1	8.7	14.2	5.6	0.47	3.94
**STR (%)**	76.1	83.0	0.0	95.0	80.0	87.0	24.9	-2.69	5.70
**LIN (%)**	57.7	62.0	0.0	80.0	58.0	67.0	19.6	-2.28	4.47
**Elong (%)**	48.9	50.0	0.0	95.0	47.0	57.0	18.4	-1.25	3.09
Area (mm*2)	4.6	5.1	0.0	8.7	4.5	5.6	1.8	-1.48	2.20

**Fig 1:**
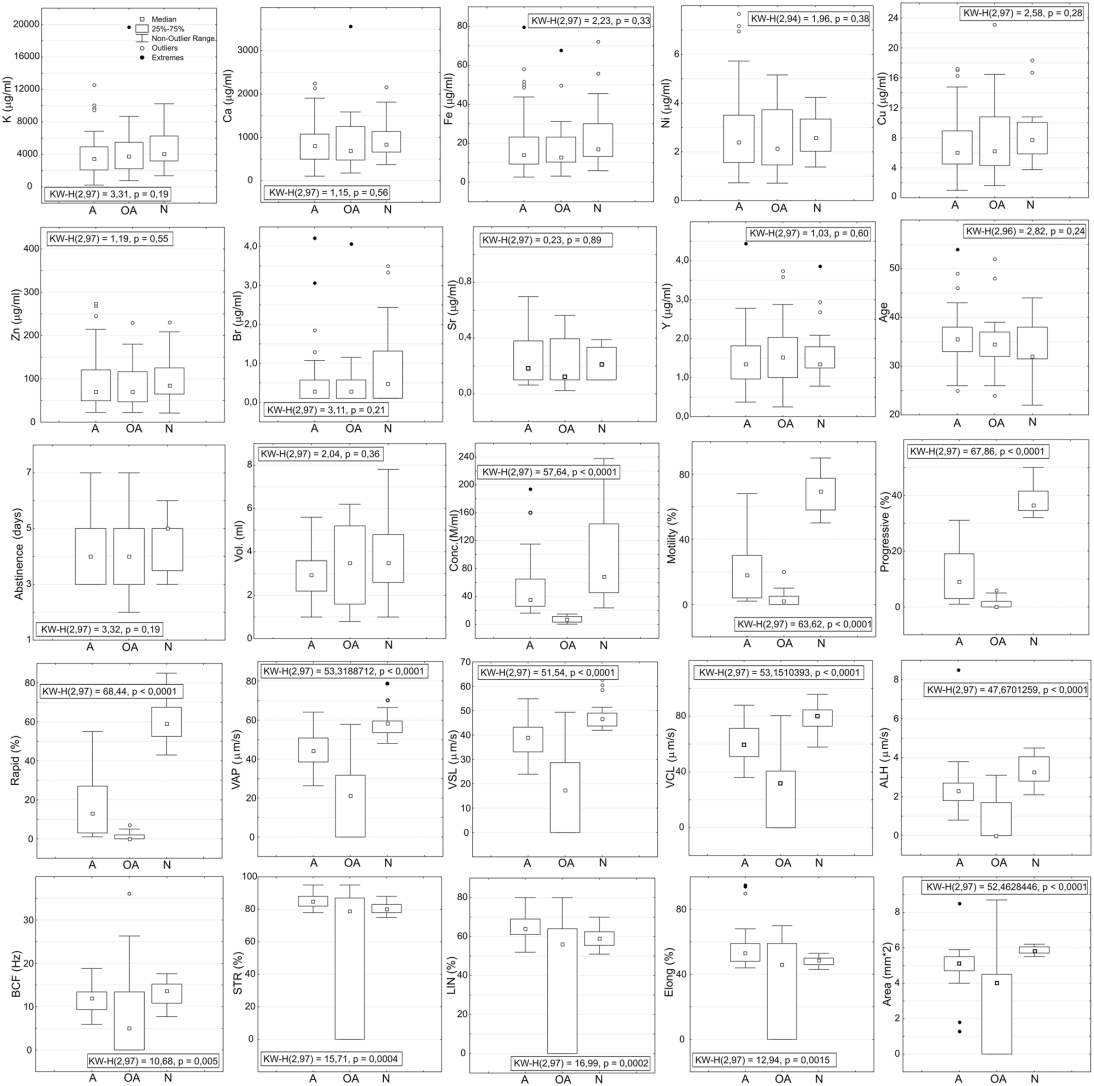
Box-plots of trace-element concentrations, patient ages, days of abstinence and sperm-quality parameters. Groups of men diagnosed with normozoospermia (N), asthenozoospermia (A) and oligoasthenozoospermia (OA) were statistically different with respect to all sperm quality parameters and sperm concentration. No significant difference was found in concentrations of chemical elements among these three groups of patients. Results of Mann-Whitney U test and Tukey HSD showed that A and N groups were statistically significantly different with respect to STR, LIN, “elongation”, VCL and ALH indicating increased expression of spontaneous spermatozoa hyperactivation among patients with normal spermiogram according to WHO criteria. The distribution of sperm quality parameters for OA group of patients was not compared to other two groups as being heavily influenced by “0” values.

Distributions of chemical elements concentration levels, age, abstinence and semen quality parameters for each diagnostic group are presented as box-plots along with results of KW test in Fig 1. Groups N, A and OA were statistically different with respect to all motility parameters and sperm concentration, but significant difference was not found in concentrations of chemical elements. Group N in comparison to group A showed lower medians of parameters STR, LIN and “elongation” and higher medians for VCL and ALH. Mann-Whitney U test and Tukey HSD showed that A and N were statistically significantly different with respect to STR, LIN, “elongation”, VCL and ALH parameters, which might be indicative of increased hyperactivation of spermatozoa among patients with “normal” spermiogram. If we define criteria for hyperactivated motility as VCL≥80 μm/s, ALH≥3.0 μm and LIN≤60%, which are similar to those defined by De Lamirande and Gagnon [5], than 40% of semen samples in normozoospermia group of patients (8 of 20) showed presence of nonlinear motility indicative of hyperactivated spermatozoa, contrary to 1.85% in asthenozoospermia group of patients (1 of 54). The distribution of sperm quality parameters for OA group of patients was not compared to other two groups as being heavily influenced by “0” values.

The possible effects of smoking on reproductive parameters were evaluated for each group of patients using the KW test. There were 4, 15 and 4 registered smokers in the N, A and OA group, respectively. Although most median values were lower in smokers, no significant differences were found between smokers and non-smokers for any group of diagnosis.

Spearman’s correlation coefficients were used to find statistically significant correlations between analysed concentration levels, age, abstinence and semen-quality parameters (Table 2). There were two groups of highly correlated data. One referred to highly positive significant correlations found between most of the measured elements (K, Ca, Fe, Ni, Cu, Zn and Y), and second referred to highly positive correlations found between sperm concentration, forward progression parameters and ALH and BCF. Correlation between K/Ca and Cu/Zn was the only that came out as statistically significant when performing correlation analysis between relative proportions of all analyzed elements. It should be noted that although K, Ca, Cu and Zn were highly positively correlated between themselves, correlation of K/Ca and Cu/Zn ratios was negative with statistical significance p = 0.01.

**Table 2:** Spearman’s correlation coefficients for 97 semen samples. Bolded correlations are significant at p < 0.05. Correlations of interest for this study are framed.

	**K**	**Ca**	**K/Ca**	**Fe**	**Ni**	**Cu**	**Zn**	**Br**	**Cu/Zn**	**Sr**	**Y**	**Age**	**Abst**.	**Vol**.	**Conc**.	**Moti**.	**Progr**.	**Rapid**	**VAP**	**VSL**	**VCL**	**ALH**	**BCF**	**STR**	**LIN**	**Elong**.
**Ca**	**0.89**	1.00																								
**K/Ca**	**0.30**	-0.10	1.00																							
**Fe**	**0.72**	**0.87**	-0.19	1.00																						
**Ni**	**0.71**	**0.80**	-0.12	**0.81**	1.00																					
**Cu**	**0.77**	**0.88**	-0.12	**0.82**	**0.80**	1.00																				
**Zn**	**0.68**	**0.66**	0.19	**0.47**	**0.47**	**0.55**	1.00																			
**Br**	0.14	0.03	**0.30**	0.01	-0.05	-0.01	0.06	1.00																		
**Cu/Zn**	0.03	0.14	**-0.26**	**0.29**	**0.29**	**0.34**	**-0.52**	-0.17	1.00																	
**Sr**	**0.26**	**0.25**	0.05	0.11	**0.24**	**0.22**	0.16	0.04	0.08	1.00																
**Y**	**0.71**	**0.74**	-0.00	**0.64**	**0.71**	**0.80**	**0.61**	0.06	0.12	**0.32**	1.00															
**Age**	-0.01	0.04	-0.12	0.02	0.06	0.06	0.03	-0.05	0.03	-0.03	0.04	1.00														
**Abst**.	0.12	0.06	0.18	-0.04	-0.04	0.03	0.14	0.01	-0.05	-0.13	0.08	0.00	1.00													
**Vol**.	-0.07	-0.14	0.12	-0.09	-0.09	-0.10	-0.19	0.15	0.09	-0.06	-0.03	-0.13	**0.29**	1.00												
**Conc**.	0.18	0.15	0.11	0.16	**0.21**	0.12	**0.20**	-0.00	-0.09	-0.11	0.06	-0.04	**0.21**	-0.08	1.00											
**Moti**.	0.05	-0.01	0.15	0.02	0.02	0.03	0.05	0.18	0.01	0.04	0.01	**-0.20**	0.19	0.07	**0.67**	1.00										
**Progr**.	0.03	-0.03	0.14	0.01	0.02	0.01	0.01	**0.20**	0.05	0.04	-0.01	-0.19	0.17	0.07	**0.65**	**0.98**	1.00									
**Rapid**	0.03	-0.02	0.13	0.02	0.01	0.01	0.01	0.13	0.03	-0.01	-0.03	**-0.22**	0.18	0.05	**0.69**	**0.97**	**0.98**	1.00								
**VAP**	-0.06	-0.12	0.11	-0.05	-0.08	-0.10	-0.11	0.15	0.04	-0.10	-0.17	**-0.22**	0.09	0.06	**0.62**	**0.82**	**0.84**	**0.87**	1.00							
**VSL**	-0.10	-0.15	0.09	-0.07	-0.10	-0.13	-0.15	0.19	0.06	-0.07	-0.19	**-0.20**	0.09	0.08	**0.56**	**0.80**	**0.84**	**0.85**	**0.98**	1.00						
**VCL**	-0.02	-0.07	0.11	-0.04	-0.04	-0.08	-0.07	0.16	0.02	-0.09	-0.15	**-0.20**	0.10	0.03	**0.64**	**0.81**	**0.82**	**0.85**	**0.97**	**0.94**	1.00					
**ALH**	0.07	-0.01	0.17	0.05	0.04	-0.02	0.00	0.09	-0.01	-0.19	-0.07	-0.13	0.18	-0.01	**0.73**	**0.76**	**0.75**	**0.79**	**0.85**	**0.80**	**0.87**	1.00				
**BCF**	0.08	-0.01	0.22	0.02	0.02	-0.06	0.05	0.15	-0.06	-0.02	-0.05	-0.14	0.17	-0.05	**0.43**	**0.34**	**0.31**	**0.37**	**0.27**	**0.25**	**0.29**	**0.36**	1.00			
**STR**	**-0.30**	**-0.23**	-0.15	-0.14	-0.10	**-0.24**	**-0.23**	0.09	0.08	0.03	-0.17	0.05	-0.05	0.03	-0.08	-0.11	-0.01	-0.06	-0.06	0.04	-0.12	-0.13	**0.20**	1.00		
**LIN**	**-0.31**	**-0.25**	-0.14	-0.16	-0.13	**-0.22**	**-0.25**	0.02	0.12	0.08	-0.18	0.07	-0.08	0.05	-0.09	-0.02	0.09	0.04	0.03	0.14	-0.05	-0.12	0.07	**0.92**	1.00	
**Elong**.	**-0.24**	**-0.21**	-0.12	-0.12	-0.18	**-0.23**	**-0.30**	-0.02	0.08	**-0.26**	**-0.34**	-0.02	-0.08	-0.02	0.01	-0.14	-0.11	-0.09	0.18	0.20	0.14	0.13	0.14	**0.52**	**0.50**	1.00
**Area**	**0.22**	0.17	0.15	0.16	0.13	0.18	**0.23**	0.15	-0.03	0.00	0.15	-0.16	**0.24**	0.05	**0.69**	**0.76**	**0.73**	**0.77**	**0.58**	**0.54**	**0.60**	**0.61**	**0.48**	-0.05	-0.04	**-0.25**

Other statistically significant correlations of importance for this research are negative correlations found for parameters STR, LIN and “elongation”, and concentration levels of K, Ca, Cu and Zn at significance level p = 0.05 to p = 0.002. It should be noted that STR, LIN and “elongation” were significantly positively correlated between themselves, but not with motility parameters VAP, VSL, VCL and ALH. Negative correlations between STR, LIN and “elongation”, and K, Ca, Cu and Zn show increase of spermatozoa nonlinear (hyperactivated) motility with increase of these elements concentrations. This was confirmed by Pearson’s correlation analysis.

**Fig 2:**
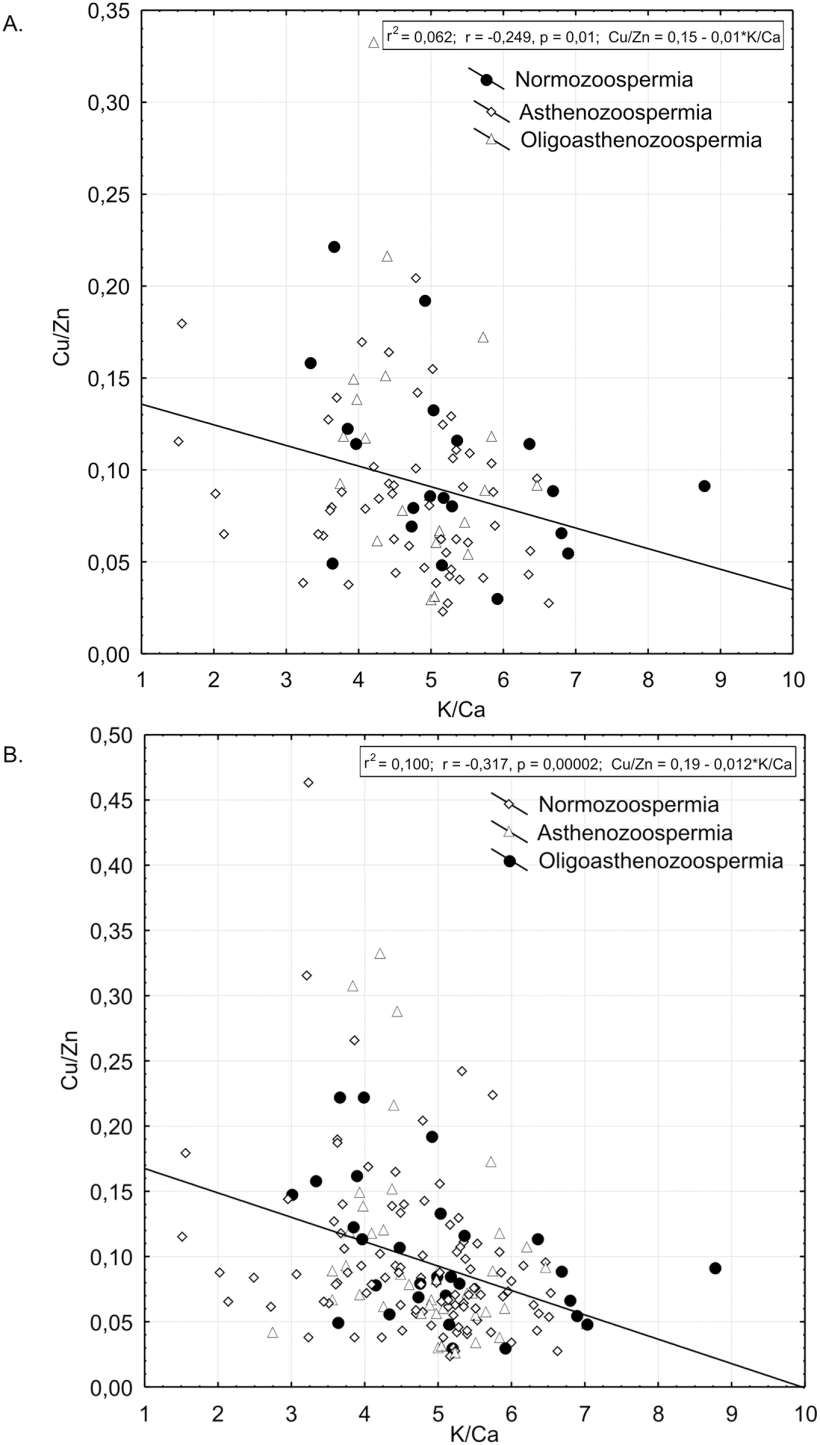
**A**. Scatter-plot and results of statistically significant linear correlation between K/Ca and Cu/Zn ratios for 97 patients recruited for this study. Samples taken from men diagnosed with normozoospermia, asthenozoospermia and oligoasthenozoospermia are distinguished by different symbols. **B**. Scatter-plot and results of statistically significant linear correlation between K/Ca and Cu/Zn ratios were checked for greater number of human semen samples. Samples taken from men diagnosed with normozoospermia (n = 30), asthenozoospermia (n = 98) and oligoasthenozoospermia (n = 47) are distinguished by different symbols. The negative correlation was confirmed to be valid for 175 semen samples at statistical significance of p = 0.00002.

**Fig 3:**
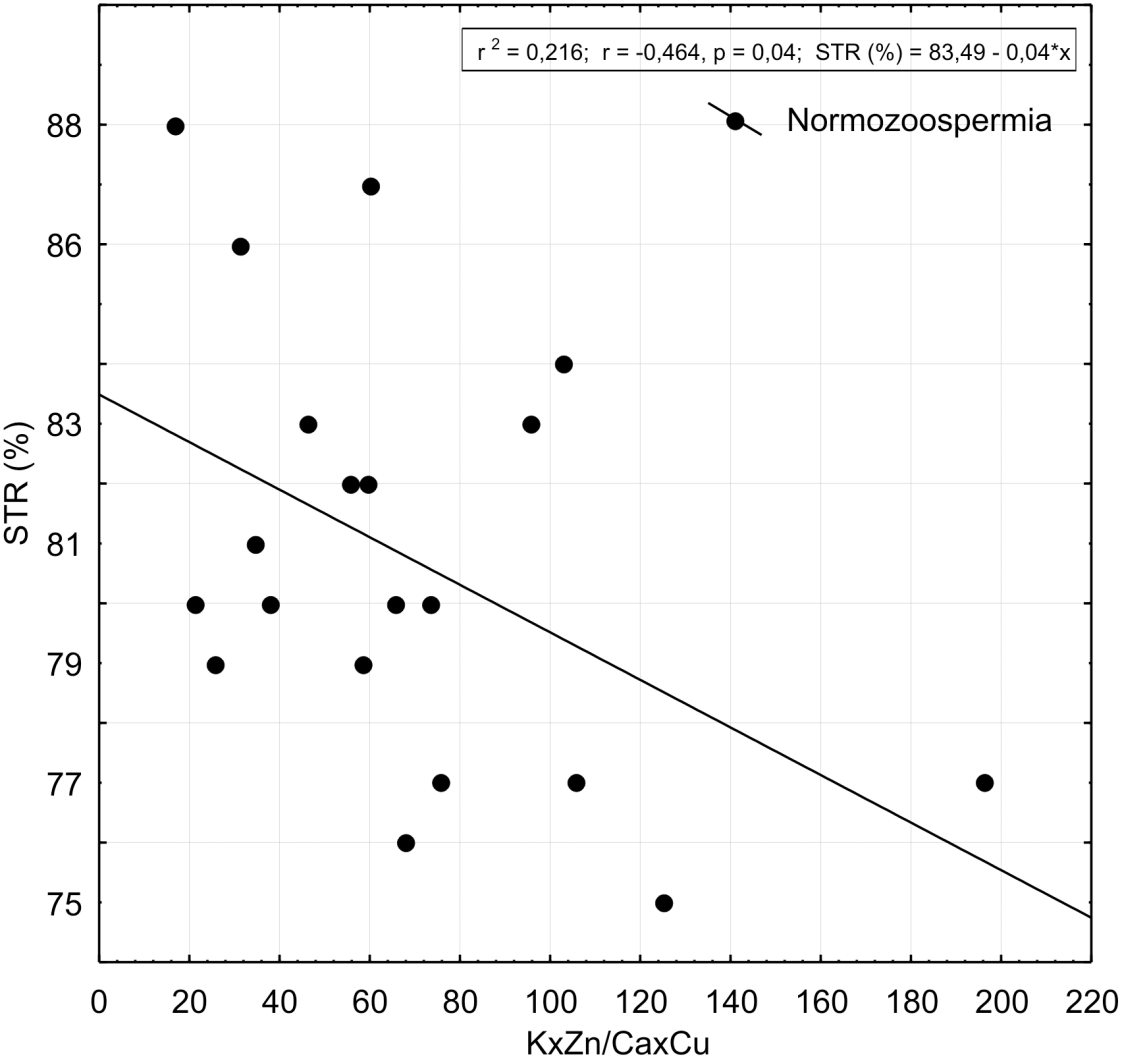
Scatter-plot and results of statistically significant linear correlation between KxZn/CaxCu ratio and parameter of spermatozoa hyperactivated motility (STR %) for samples taken from the group of men with normal spermiogram, although with expressed spontaneous spermatozoa hyperactivation. Increased concentrations of K and Zn with respect to Ca and Cu are related to low STR values revealing synergistic effects of these elements in relation to presence of spontaneous hyperactivation of spermatozoa.

Regression analyses of K/Ca and Cu/Zn ratios for 97 semen samples of patents involved in this study is presented in Fig 2a. We tested if the negative correlation between K/Ca and Cu/Zn ratio works for greater number of semen samples. We found the negative correlation to be valid for 175 semen samples at statistical significance of p = 0.00002 (Fig 2b). Fig 3 shows regression analysis between K/Ca and Cu/Zn ratio and STR in group of patients with normal spermiogram according to WHO criteria, but with indication of increased spontaneous spermatozoa hyperactivation in comparison to patients with asthenozoospermia.

### Principal Component Analysis

**Fig 4:**
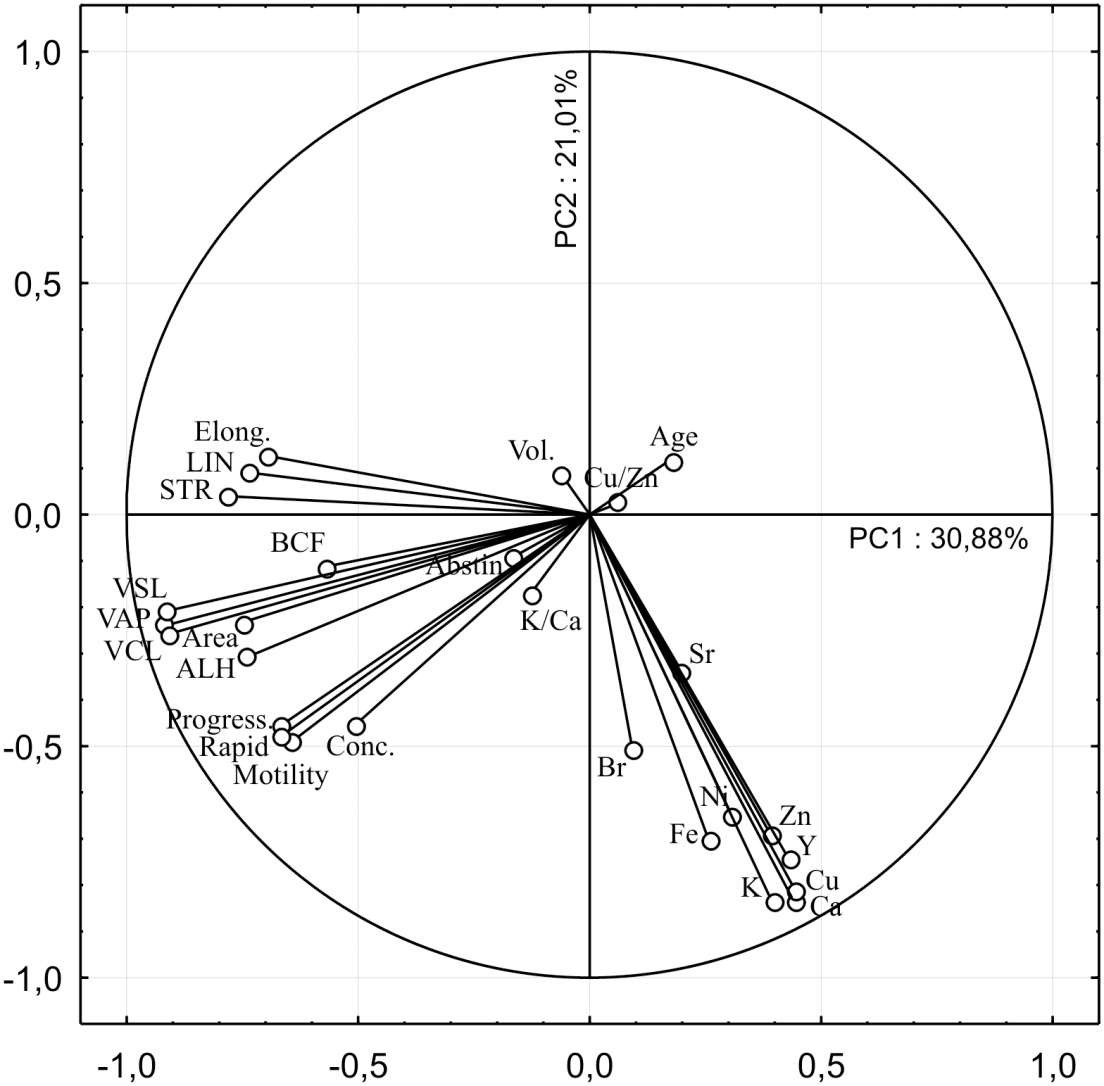
Graphical display of variables contributing to principal component 1 (PC1) and principal component 2 (PC2) and amount of total variance explained per each PC showing strong association of sperm quality parameters including sperm concentration around PC1 and concentration of elements around PC2. The variables concentrated around graphical centre do not contribute significantly to these two PCs, although they could be interpreted by subsequent PCs describing remaining data variance. The projection of K, Ca, Cu, Zn and Y variables on PC1 describing 31% of total variance shows weaker negative correlation to motility parameters and sperm concentration. Higher concentrations of these elements negatively impact forward progression parameters and sperm concentration, but contribute to hyperactivation motility expressed as low STR and LIN, accompanied with low “elongation” (a morphological parameter). This also implies that hyperactivation activates on expense of forward progression. PC2 describing 21% of remaining variance shows that all elements are correlated between themselves although Br and Sr express lower contribution. Their higher concentrations are associated to greater forward progression and sperm concentration. These results imply that, although of secondary importance, the higher baseline status of elements improves forward progression and sperm concentration.

PCA was used to reduce the numerous analysed variables to a smaller number of orthogonal factors called principal-component axes (PCs). The variables that contribute the most variance to a selected PC are correlated, suggesting that their distributions may be controlled by a common process. Extracted PCs are linearly uncorrelated and define the subspace containing most of the variation of the initial data matrix, where every subsequent axis describes most of the remaining variance. We chose two first PCs for interpretation explaining 51.89% of the total variance (Fig 4). The absolute contributions of variables to selected PCs are presented in Table 3.

**Table 3:** The absolute contributions of variables to selected PC axes; (marked loadings ≥ 0.35).

	PC1	PC2
**Kμg/ml**	**0.39**	**-0.84**
**Ca μg/ml**	**0.44**	**-0.83**
**Fe μg/ml**	0.26	**-0.70**
**Ni μg/ml**	0.31	**-0.65**
**Cu μg/ml**	**0.44**	**-0.81**
**Zn μg/ml**	**0.39**	**-0.69**
**Br μg/ml**	0.09	**-0.51**
**Sr μg/ml**	0.20	-0.34
**Y μg/ml**	**0.43**	**-0.74**
**K/Ca**	-0.13	-0.17
**Cu/Zn**	0.06	0.03
**Age**	0.18	0.12
**Abst. (days)**	-0.17	-0.09
**Vol. (ml)**	-0.06	0.09
**Conc.(M/ml)**	**-0.51**	**-0.45**
**Motil.(%)**	**-0.65**	**-0.49**
**Progres. (%)**	**-0.67**	**-0.45**
**Rapid (%)**	**-0.67**	**-0.48**
**VAP (mm/s)**	**-0.92**	-0.24
**VSL (mm/s)**	**-0.92**	-0.21
**VCL (mm/s)**	**-0.91**	-0.26
**ALH (mm/s)**	**-0.74**	-0.31
**BCF (Hz)**	**-0.57**	-0.11
**STR (%)**	**-0.79**	0.04
**LIN (%)**	**-0.74**	0.09
**Elong (%)**	**-0.69**	0.13
Area (mm*2)	**-0.75**	-0.23
**Explaind Variance**	8.3373	5.6731
**Proportional of Total**	0.3088	0.2101

PC1, which described 30.88% of total variance, confirmed results of Spearman analysis in terms of association of high K, Ca, Cu and Zn concentrations to increased hyperactivated spermatozoa. It also detected Y as additional element which might play a role in hyperactivation. Furthermore, PC1 showed that higher concentrations of K, Ca, Cu, Zn and Y negatively impact forward progression parameters and sperm concentration. Thus, it can be concluded that spermatozoa hyperactivated motility comes on expense of forward progression. PC2, which described 21.01% of the remaining variance, showed that although of secondary importance, the higher baseline status of all measured elements except Sr, improves forward progression and sperm concentration.

## Discussion

KW analysis did not show significant difference in concentrations of chemical elements among normozoospermia, asthenozoospermia and oligoasthenozoospermia groups of patients. These results were not influenced by age, smoking or exposure to toxic chemical elements. Thus, human semen samples showing low or high baseline status of chemical elements concentrations can be found in samples from all three groups of patients. Although inadequate concentration levels of analysed elements obviously are not the limiting factor for sperm concentration and forward progression, which are diagnostic sperm quality parameters for asthenozoospermia (poor progressive motility) and oligoasthenozoospermia (poor progressive motility and low sperm count), baseline concentration status could be of some importance as shown by PCA-Principal Component 2.

Both analyses, Spearman’s correlation and PCA, showed that increased concentrations of K, Ca, Cu and Zn were associated with low values of parameters LIN and STR, indicative of increased spermatozoa hyperactivation. In addition to low values of LIN and STR, which are generally recognized as hyperactivated sperm characteristics, our results showed that low “elongation”, i.e. thinner sperm head, was also indicative of hyperactivation. Expression of hyperactivated motility came on expense of forward progression as shown by PC1.

The composition of the external spermatozoa environment has profound effects on spermatozoa transit [27]. Spermatozoa transit involves two general forms of motility, progressive and hyperactivated, whereas activation of spermatozoa progressive motility is a prerequisite for occurrence of hyperactivation.

Extracellular K ions in alkaline media and controlled production of ROS may give rise to different patterns of Ca ion influx by regulating sperm membrane permeability. Ca ion influx regulates activation of progressive motility, however higher Ca-influx triggers hyperactivated motility [28] and even arrest spermatozoa [1]. It was showed that spermatozoa arrest when exposed to very high levels of extracellular K ions, either because of Ca overload [1] or because of K induced inhibition of enzymes responsible for fructolysis [25]. Concentrations of Zn, ROS production and activity of CuZnSOD, concentration of extracellular K and Ca-ion influx are closely regulated in semen in order to provide the right balance for adequate motility status. The negative correlation found between K/Ca and Cu/Zn ratio in semen showed by this study may be explained by different proportions of male reproductive tract secretions (presumably from prostate and seminal vesicles) affecting the levels of K, Ca, Cu and Zn concentrations in analyzed ejaculates. Furthermore, given that the same correlation can be read as K x Zn (abscise) in relation to Cu x Ca (ordinate), it could be assumed that Cu and Ca as well as K and Zn may be interconnected through the same processes or even involved in functions of the same enzymes participating in sperm motility induction.

Our results show that higher concentrations of K and Zn with respect to Ca and Cu are related to lower values of STR parameter in semen samples of men with normal spermiogram, revealing synergistic effects of these elements in relation to spontaneous hyperactivation of spermatozoa. These results are in accordance to research conducted by Sørensen *et al*. [29] who found that men from healthy couples (based on conventional semen and CASA parameters) who had short time-to-pregnancy (1–2 months) had lower Zn/Ca ratio median in semen samples compared to men from couples who had long time-to-pregnancy (7–28 months) [29]. Semen samples showing Zn/Ca ratio above 0.22 had statistically significant lower values for the CASA parameters VSL, LIN and STR compared to those samples with a ratio below 0.22. Thus the authors showed that higher Zn concentrations and lower Ca concentrations in semen samples of healthy men were related to increased number of hyperactivated spermatozoa in ejaculate, which as a consequence took longer time for couples to conceive.

Availability of K, Zn, Ca and Cu ions at the right time and in right concentrations is very important for sperm to obtain and maintain its motility. At ejaculation sperm is expelled in first third of ejaculate [12] together with prostatic fluid rich in K, Ca and Zn [9]. The possible role of Zn in the prostatic fluid may be to protect sperm from shear forces at ejaculation by preserving its soft chromatin [12] and ODF structures. After ejaculation zinc from chromatin and ODF is rapidly lost, probably by adding last two thirds of ejaculate containing seminal vesicular fluid, which is more alkaline and rich in chelating agents [12, 14] and CuZnSOD [8]. It was observed that chelation of Zn ions induces sperm progressive motility [14, 30]. Sørensen *et al*. [29] found that zinc-rich seminal plasma made the sperm cells moving in a more random and less forward fashion, but the number of motile sperm cells was not reduced. The role of seminal vesicular fluid is likely to enable progressive sperm motility and to prevent activation of premature sperm hyperactivation in ejaculated semen by chelation of K, Ca and Zn and by activation of CuZnSOD.

Sperm hyperactivation induced by Ca ion influx is promoted by membrane depolarization in media rich in extracellular K ions [21] (and other depolarizing agents), or by membrane hyperpolarization through the activation of Slo3 channels [3, 23], possibly induced by Zn ions [18]. Both processes require alkaline media. Lishko *et al*. [19] showed that removal of external Zn^2+^ activates Hv1, a human specific proton channel which activates through membrane depolarization. These findings impose that membrane depolarization and hyperpolarization may substitute or complement one another in increasing pHi, as noted by Chavez *et al*. [23]. The mechanisms showed to be controlled by external K (membrane depolarization) and Zn ions (membrane hyperpolarization). Our studies confirm that excessive Zn and K ions, especially in the presence of insufficient concentrations of CuZnSOD and Ca, act together in promoting sperm hyperactivation.

Seminal vesicular fluid contains higher concentration of antioxidants than any other biological fluid [8]. CuZnSOD is one of the main semen enzymes for protecting spermatozoa from overproduction of ROS [8]. Semen with lower scavenging capacity than normal has much higher values of spontaneous hyperactivation [13]. De Lamirande & Gagnon [5] found that 18% of semen samples from patients whose spermiograms were considered normal according to the WHO criteria expressed spontaneous hyperactivation. The ROS scavenging capacity of seminal plasma and spermatozoa from these patients were, respectively, 37% and 40% lower than those found in seminal plasma and spermatozoa from normal men. Insufficient seminal Cu is most likely indicative of insufficient CuZnSOD concentration, which in turns causes overproduction of ROS and consequently spontaneous hyperactivation. Presence of increased concentrations of seminal Zn may inhibit the CuZnSOD activity [17] at the same time preventing the stiffening of sperm strucural fibers resulting in premature hyperactivation [12]. Therefore, extracellular Zn in semen may have several roles in promoting sperm hyperactivated motility, either by enabling processes of Ca influx or by being reversible incorporated in the spermatozoa ODF.

Extracellular Ca is necessary for sperm to obtain motility [3]. In this research it was showed that decreased availability of Ca in presence of excessive Zn and K and decreased concentrations of CuZnSOD promotes sperm hyperactivation. There are observations showing the role of extracellular Ca ion may be to increase CuZnSOD activity against ROS [31], which would prevent spermatozoa hyperactivation. Thus Ca-ion may have dual role in promoting hyperactivation (by Ca ion influx through activated CatSper channels as a consequence of increased pHi) and preventing spermatozoa hyperactivation (by increasing CuZnSOD activity).

Although PCA analysis indicated that Y can also contribute to hyperactivation, its role was not investigated in this study. It is known, however, that Y enters the sperm flagellum [32] and binds at Ca sites with greater affinity than Ca ions [32]. Coomes [33] found that radioactive ^90^Y was specifically taken up from a ^90^Sr/^90^Y mixture by rat spermatozoa flagellum, but not by the sperm heads.

## Conclusions

Although this survey founds no statistically significant difference in the distribution of concentrations of chemical elements in semen samples from men diagnosed with normozoospermia, asthenozoospermia and oligoasthenozoospermia, baseline concentration status could be of some importance as shown by PCA—Principle Component 2. Statistically significant difference was found between increased activation of hyperactivated motility (decreased values of STR, LIN and morphological parameter “elongation”) and increased concentrations of elements K, Ca, Cu and Zn. This was confirmed by PCA—Principal Component 1, which also showed that spermatozoa hyperactivation comes on expense of progressive motility. The negative correlation was found between K/Ca and Cu/Zn. Synergistic effects of K, Ca, Cu and Zn were evident from relation showing higher concentrations of K and Zn with respect to Ca and Cu to promote spontaneous spermatozoa hyperactivation. This was found in group of patients with “normal” spermiogram, who were showing higher spontaneous spermatozoa hyperactivation compared to asthenozoospermia group of patients. This also indicates that sperm quality of patients with normal spermiogram according to WHO criteria may be compromised by showing spontaneous hyperactivation which could be a sign of impaired sperm function as a consequence of seminal elemental composition imbalance. These findings emphasize the importance of evaluation of multi-elemental synergistic effects in semen which are rarely studied.
